# Comparison of the Dielectric Properties of Ecoflex^®^ with L,D-Poly(Lactic Acid) or Polycaprolactone in the Presence of SWCN or 5CB

**DOI:** 10.3390/ma14071719

**Published:** 2021-03-31

**Authors:** Patryk Fryń, Sebastian Lalik, Natalia Górska, Agnieszka Iwan, Monika Marzec

**Affiliations:** 1 Institute of Physics, Jagiellonian University, S. Lojasiewicza 11, 30-348 Krakow, Poland; patryk.fryn@doctoral.uj.edu.pl (P.F.); sebastian.lalik@doctoral.uj.edu.pl (S.L.); 2 Faculty of Chemistry, Jagiellonian University, Gronostajowa 2, 30-387 Krakow, Poland; gorska@chemia.uj.edu.pl; 3 Faculty of Security and Safety Research, General Tadeusz Kosciuszko Military University of Land Forces, Czajkowskiego 109 Str., 51-147 Wroclaw, Poland

**Keywords:** dielectric spectroscopy, FTIR, single walled carbon nanotubes, L,D-poly(lactic acid), polycaprolactone, Ecoflex^®^, 5CB, oleic acid

## Abstract

The main goal of this paper was to study the dielectric properties of hybrid binary and ternary composites based on biodegradable polymer Ecoflex^®^, single walled carbon nanotubes (SWCN), and liquid crystalline 4′-pentyl-4-biphenylcarbonitrile (5CB) compound. The obtained results were compared with other created analogically to Ecoflex^®^, hybrid layers based on biodegradable polymers such as L,D-polylactide (L,D-PLA) and polycaprolactone (PCL). Frequency domain dielectric spectroscopy (FDDS) results were analyzed taking into consideration the amount of SWCN, frequency, and temperature. For pure Ecoflex^®^, two relaxation processes (α and β) were identified. It was shown that the SWCN admixture (in the weight ratio 10:0.01) did not change the properties of the Ecoflex^®^ layer, while in the case of PCL and L,D-PLA, the layers became conductive. The dielectric constant increased with an increase in the content of SWCN in the Ecoflex^®^ matrix and the conductive behavior was not visible, even for the greatest concentration (10:0.06 weight ratio). In the case of the Ecoflex^®^ polymer matrix, the conduction relaxation process at a frequency ca. several kilohertz appeared and became stronger with an increase in the SWCN admixture in the matrix. Addition of oleic acid to the polymer matrix had a smaller effect on the increase in the dielectric response than the addition of liquid crystal 5CB. Fourier transform infrared (FTIR) results revealed that the molecular structure and chemical character of the Ecoflex^®^ and PCL matrixes remained unchanged upon the addition of SWCN or 5CB in a weight ratio of 10:0.01 and 10:1, respectively, while molecular interactions appeared between L,D-PLA and 5CB. Moreover, adding oleic acid to pure Ecoflex^®^ as well as the binary and ternary hybrid layers with SWCN and/or 5CB in a weight ratio of Ecoflex^®^:oleic acid equal to 10:0.3 did not have an influence on the chemical bonding of these materials.

## 1. Introduction

Among the important topics in recent science, conductive biodegradable materials can be distinguished, considering their use as flexible electrodes in optoelectronic devices such as solar cells, light-emitting diodes, or transistors [1,2,3,4,5,6,7,8,9,10,11,12]. Skillful highlighting of the selected properties of individual components of the produced inorganic–organic compositions may allow for the construction of an innovative electrode based on unique solutions. Another essential aspect that should be taken into account is the analysis of the environmental impact of the materials used in terms of the principles of green (safe) chemistry and sustainable development. The ecological problems that we are currently encountering worldwide do not allow us to stand idly by, and therefore, we want to take care of the planet in accordance with the principles of BAT (best available techniques) as part of our research.

For this reason, we investigated hybrid layers based on biodegradable polymers, liquid crystal, and single-wall carbon nanotubes (SWCN). In our previous research [13,14], we developed for the first time three-component composite materials based on biodegradable polymer L,D-polylactide (L,D-PLA), conductive SWCN, and liquid crystal 4-pentyl-4′-cyanobiphenyl (5CB) for use as an electrode in opto-electronic devices. Comprehensive physico-chemical analysis of the created compositions allowed for an innovative conclusion that the liquid crystalline component (5CB) increased the flexibility of the material and, most importantly, increased the conductivity. The best parameters, in terms of durability and conductivity, were obtained for the L,D-PLA:5CB:SWCN composite material with a mutual ratio of 10:1:0.5 *w*/*w*/*w*. An additional success was to obtain a partially transparent layer exhibiting electrical conductivity. The development of such a layer gives hope that further optimization will allow the creation of a layer with even greater transparency as the electrical conductivity increases. This is important because the global resources of indium, used for the production of indium tin oxide (ITO), commonly used as a transparent electrode in opto-electronic devices, are running low, which significantly affects its increasingly higher price. Therefore, the search for cheaper alternatives to ITO is one of the challenges science is currently facing.

Moreover, we have recently created three-component composite materials based on biodegradable polymers, polycaprolactone (PCL), and Ecoflex^®^ with SWCN and 5CB admixtures for application as an electrode in opto-electronic devices [15]. For the first time, using atomic force imaging (AFM), we have shown that PCL and Ecoflex^®^ polymers visibly cover carbon nanotubes. On the other hand, the SWCN are in contact with themselves when trapped in the L,D-PLA matrix. Such a difficult contact in the case of PCL and Ecoflex^®^ polymers affects their electrical properties.

Considering the operation of the opto-electronic devices, the electric field effect should be taken into account. Permittivity is a physical quantity that describes how an electric field affects and is affected by a dielectric medium and it is determined by the ability of a material to polarize in response to an applied electric field, and thereby to partially cancel the field inside the material. As is well known, the dielectric anisotropy is expressed as Δε = ε_||_ − ε_⊥_, where ε_||_ and ε_⊥_ are the parallel and perpendicular components of the electric permittivity, respectively [16].

Some researchers have studied the dielectric properties of biodegradable polymers such as PCL or polylactide (PLA). For example, Dichtl et al. [17] focused on the dielectric properties of a commercially available PLA filament that was printed via fused filament fabrication in a 3D printer. The dielectric analyses revealed dipolar relaxation processes and a significant change in the dielectric properties during cold crystallization. The plateau of the main relaxation was proportional to the degree of crystallization. Moreover, the authors demonstrated that the conductivity of PLA could be enhanced by mixing it with the ionic liquid trihexyl tetradecyl phosphonium decanoate. On the other hand, Ren et al. [18,19,20] investigated the thermal and dielectric behavior of blends consisting of semicrystalline poly(L-lactic acid) and amorphous poly(DL-lactic acid). The PLA samples were amorphous and exhibited three dielectric loss peaks designated as α’, α, and β in the order of decreasing temperature. The dielectric properties of DL-PLA were also reported by Mierzwa et al. [21] and Mijovic and Sy [22]. The effect of crystallinity on the dielectric relaxation α in semicrystalline L-PLA with various degrees of crystallization was studied and it was found that the crystallization did not affect the relaxation time of the α process [22]. On the other hand, Bras et al. [23] used dielectric spectroscopy to study the crystallization of PLA and found that the crystallization could be described by a linear combination of three processes with different intensities: the α-process of the amorphous material, the constrained α-process present in the fully crystallized material (corresponding to the segmental motions of the amorphous phase confined by the crystalline lamellae), and the sub-glass β-relaxation. Moreover, the dielectric properties of PLA/chitosan nanocomposites were investigated by Elsawy et al. [24], where the α-relaxation process was analyzed with Vogel–Fulcher–Tamman and Havriliak–Negami models. It was shown that the dielectric spectra of the amorphous samples exhibited two main α relaxation processes for the fully amorphous phase, while the process appearing at relatively high temperature was assigned to the constrained amorphous phase between crystalline lamellae. The intensity of the ε” maxima of both processes increases with an increase in the content of chitosan nanoparticles, but their positions are weakly dependent on the composition of the nanocomposites. Interesting work was done by Spinelli et al. [25] where multiwalled carbon nanotubes (MWCNTs), graphene nanoplates (GNPs), or a combination of both up to 12 wt% were used for producing 3D-printed specimens based on PLA. They showed that for PLA enriched with 12 wt% of MWCNTs, the relative permittivity reached the value of 5.35 × 10^3^, which was much higher than 3.7 for unfilled PLA.

Significant work was also conducted by several researchers to investigate the dielectric properties of PCL. For example, the dielectric relaxation spectra of poly(ε-caprolactone) networks hydrophilized by copolymerization with 2-hydroxyethyl acrylate (HEA) were studied by Serra et al. [26]. It was found that the α-relaxation for this specific PCL shifted to higher temperatures compared to the linear (uncrosslinked) polymer. It turns out that the incorporation of hydrophilic units in the network involves a new secondary mode (β) due to the association of side HEA chains through hydrogen bonding with water molecules. Moreover, the intensity of this relaxation increases with the amount of HEA in the investigated composition [26]. On the other hand, dielectric properties of polystyrene/polycaprolactone composites prepared by miniemulsion polymerization were studied by Ibrahim et al. [27], while Ravi et al. [28] investigated the electrical properties of biodegradable poly(ε-caprolactone):lithium thiocyanate complexed polymer electrolyte films. The authors found that the dielectric permittivity reached a higher value at a lower frequency and that it increased with increasing temperature due to the polar nature of the host polymer.

Regarding Ecoflex^®^, to the best of our knowledge, the dielectric properties of Ecoflex^®^ have not been investigated yet, however, scientists have analyzed other properties of Ecoflex^®^ and their various hybrid compositions toward practical use. For example, composites of Ecoflex^®^ with carbon nanotubes have been tested in supercapacitors and skin-mountable strain sensors [29,30,31,32,33]. Interesting work was presented by Luis et al. [34] where mechanical properties of Ecoflex^®^ was widely investigated as a very promising material for human silicone implants. Compositions based on Ecoflex^®^ and graphene oxide were investigated in our previous work toward organic photovoltaics [9].

In this work, we investigated the dielectric behavior of three biodegradable polymers such as Ecoflex^®^, L,D-poly(lactic acid), and polycaprolactone doped with SWCN or 5CB as a function of temperature frequency, and the added amount of SWCN. Binary and ternary hybrid layers were created and investigated in detail. The dielectric permittivity and tangent delta were analyzed, taking into consideration the amount of SWCN and temperature. To the best of our knowledge, this is the first paper describing in detail the dielectric properties of hybrid layers based on Ecoflex^®^ doped with SWCN or 5CB. Additionally, the proper chemical compositions and influence of oleic acid, SWCN, and 5CB on chemical bonding in the studied polymers were investigated using ATR FTIR spectroscopy.

## 2. Materials and Methods

### 2.1. Materials

L,D-poly(lactic acid) (L,D-PLA) was used as received from Galactic. Biodegradable aliphatic-aromatic copolyester (under the commercial mark Ecoflex^®^ produced by BASF, Ludwigshafen, Germany) was used as received. The polycaprolactone (PCL), 4′-pentyl-4-biphenylcarbonitrile (5CB), and single walled carbon nanotubes (SWCN) were used as received from Sigma-Aldrich (Saint Louis, Missouri, USA). The SWCN used had an average diameter of 0.84 nm, median length 1 µm, ≥95% carbon basis (≥99% as carbon nanotubes). Chloroform and oleic acid were used as received from POCH.

Ecoflex: FT-IR [cm^−1^]: 726 δ_oop_(CH)_ar_; 750 ρ(CH_2_)_n_; 795, 806, 812, 873 δ_oop_(CH)_ar_; 916, 935; 1017, 1028 δ_ip_(CH)_ar_; 1104, 1119, 1165 ν(O–C–C); 1251, 1268 ν(C–C–O); 1320, 1364, 1390 δ_s_(CH)_al_; 1410, 1452 δ_as_(CH)_al_; 1459, 1504, 1578 ν(C=C)_ar_; 1709 ν(C=O)_ar_, 1726 ν(C=O)_al_; 2871, 2897, 2958 ν(CH)_al_; 3053 ν(CH)_ar_ cm^−1^.

PCL: FT-IR [cm^−1^]: 709, 727, 732 ρ(CH_2_)_n_; 840, 934, 961, 1045, 1066, 1093 sh, 1107; 1177, 1186 ν_s_(OC–O); 1239 ν_as_(C–O–C); 1293 ν_cr_(C–O) and ν_cr_(C–C); 1354 sh, 1366, 1373 sh, 1397 δ_s_(CH); 1418, 1436, 1458, 1464, 1470, 1476 δ_as_(CH); 1722 ν(C=O); 2864 ν_s_(CH); 2895; 2944 ν_as_(CH) cm^−1^.

5CB: FT-IR [cm^−1^]: 565 δ_oop_(ring)_ar_; 637, 650; 724 ρ(CH_2_)_n_; 809, 829, 856 δ_oop_(CH)_ar_; 967 br; 1006, 1024 δ_ip_(CH)_ar_; 1111, 1119, 1127; 1180, 1186 δ_ip_(CH)_ar_; 1286, 1378, 1397 δ_s_(CH)_al_; 1455, 1466 δ_as_(CH)_al_; 1494, 1605 ν(C=C)_ar_; 2226 ν(C≡N); 2856, 2869, 2927, 2955 ν(CH)_al_; 3026, 3070 ν(CH)_ar_ cm^−1^ (br—broad, sh—shoulder, ρ—rocking, ip—in plane, oop—out of plane, ar—aromatic, al—aliphatic, n—chain, cr—in crystalline phase, s—symmetric, as—asymmetric).

The IR band positions of L,D-PLA have previously been presented [14].

### 2.2. Preparation of Hybrid Materials

All fabricated hybrid layers with various additives were prepared in the same four step procedure, as is presented briefly below:

1. The appropriate amount of polymer (ca. 0.5–1.5 g per layer) was poured into chloroform (8 mL chloroform per 1 g of L,D-PLA or PCL and 4 mL per 1 g of Ecoflex^®^).
2. The admixture (SWCN and/or 5CB and/or oleic acid) was added in the proper weight ratio.
3. The solution was mixed with a magnetic stirrer for at least 2 h and followed by sonication (Ultrasonic Processors—VCX500, tip 2 mm) for 40 min, 10 min, and 20 min for L,D-PLA, PCL, and Ecoflex^®^, respectively.
4. The mixture was poured onto an 8 cm diameter glass substrate to form a flexible film after evaporating the chloroform under a fume cupboard.

It should be noted here that the creation of a continuous layer based on Ecoflex^®^ at the same condition as the other two biopolymers is quite difficult and peeling off from the substrate damages the layer. In the case of thicker layers (over 150 micrometers), the polymer spontaneously detaches from the substrate as the solvent evaporates, but crumbles into many fine flakes. It turned out, however, that the appropriate proportion of chloroform to the polymer (6 mL of chloroform to 1.5 g of Ecoflex^®^, Petri dish with a diameter of 8 cm) allowed us to obtain larger continuous layers with a diameter of more than 10 mm and thus it was possible to carry out dielectric measurements in the same way for all tested biodegradable polymers. Taking into account the above limitation, the maximum addition of SWCN to the Ecoflex^®^ matrix was 0.06:10 (at a higher concentration of SWCN, a very sticky mixture was formed, which was difficult to pour into a Petri dish).

### 2.3. Characterization of Methods

**Figure 1:**
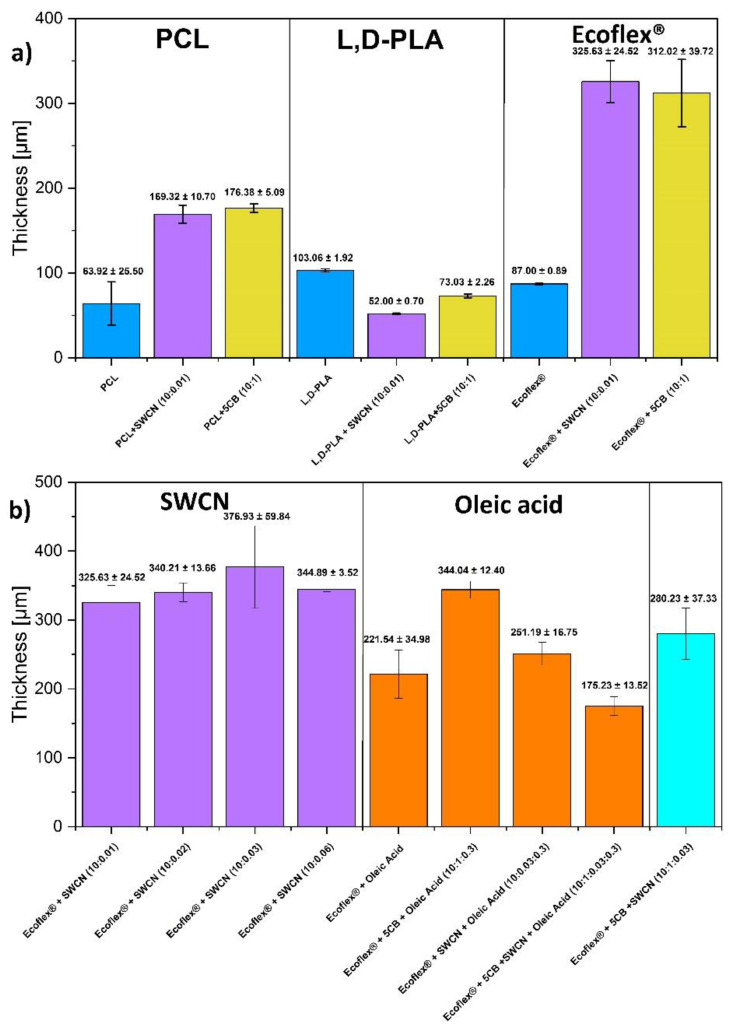
Thickness of the created layers of PCL, L,D-PLA and Ecoflex® without an with SWCN or 5CB admixture (**a**) and Ecoflex® with different admixtures (**b**). The standard errors are given.

The frequency domain dielectric spectroscopy (FDDS) method was used to study the electric properties of the new created hybrid layers. For this purpose, the Turnkey Impedance Spectrometer Concept 81 (Novocontrol Technologies GmBbH & Co. KG, Montabaur, Germany) was used. Measurements were performed versus temperature between 0 °C and 50 °C in the frequency range from 1 Hz to 1 MHz at a measuring oscillation voltage of 0.5 V in the plate capacitor geometry for the thin roundel cut from the created layers (diameter of 10 or 20 mm, thickness of c.a. 50–350 µm, depending on the hybrid material, see Figure 1).

Texture observations were performed using a Nikon Eclipse LV100POL polarizing microscope with bright-field illumination. The images were registered at room temperature by the computer-controlled camera Canon EOS 600D with and without an analyzer. A small piece of each created hybrid layer (c.a. 1 cm^2^) was placed between the glass substrate and the cover glass.

ATR FTIR spectra were collected using a Thermo ScientificTM NicoletTM iS5 spectrometer equipped with an iD5 ATR accessory (Thermo Fisher Scientific, MA, USA). Spectra of created layers were obtained at room temperature in the range of 550–4000 cm^−1^, with a spectral resolution of 2 cm^−1^ and at 32 scans per spectrum. Before the measurement of each spectrum, the background spectrum was collected to avoid air impurities. The spectra were registered using OMNIC 8.3 software whereas full spectrum processing was performed using Bruker Opus 7.0.

**Figure 2:**
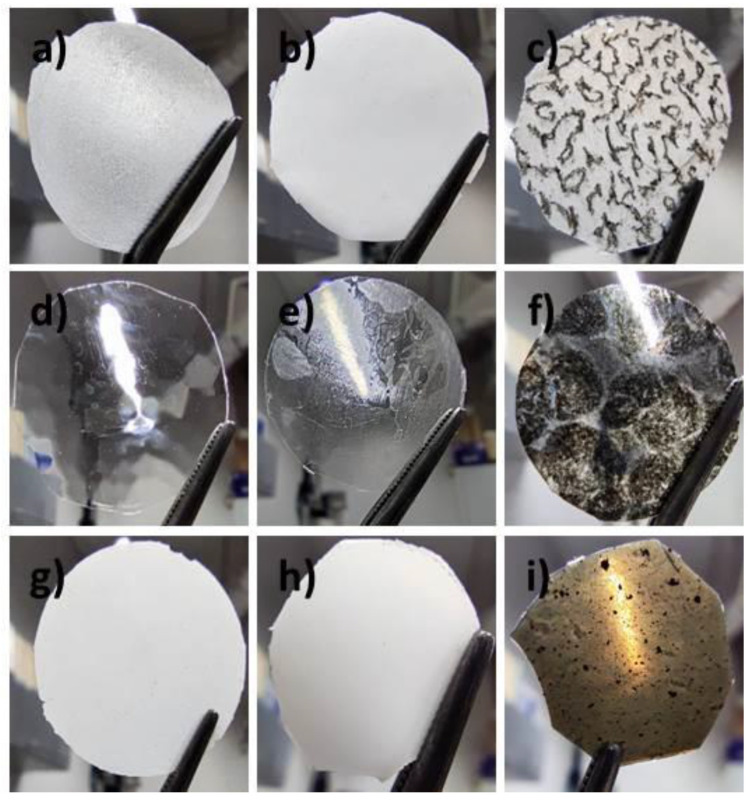
Created hybrid layers: PCL (**a**), PCL:5CB (**b**), PCL:SWCN (**c**); L,D-PLA (**d**), L,D-PLA:5CB (**e**), L,D-PLA:SWCN (**f**); Ecoflex^®^ (**g**), Ecoflex^®^:5CB (**h**), Ecoflex^®^:SWCN (**i**).

Thickness of the created hybrid layers was determined using a DektakXT (Bruker Co., Billerica, MA, USA) Stylus profilometer working in N-Lite mode which ensures a very low stylus load (radius 2 µm, load 0.3 mg) on the sample surface, assuring a nondestructive way of analysis. Layers were placed on the glass substrate and the profiles of the cross section were measured, while the ground level was set on a glass substrate. Thickness was determined by the difference between the average profile height and the substrate level. The thickness of the created hybrid layers are shown in Figure 1 while their photos are presented in Figure 2 and in Figure S1 in Supplement. As can be seen, the homogeneity and transparency of the created layer depends on the polymer and dopant used.

### 2.4. Oleic Acid

**Figure 3:**
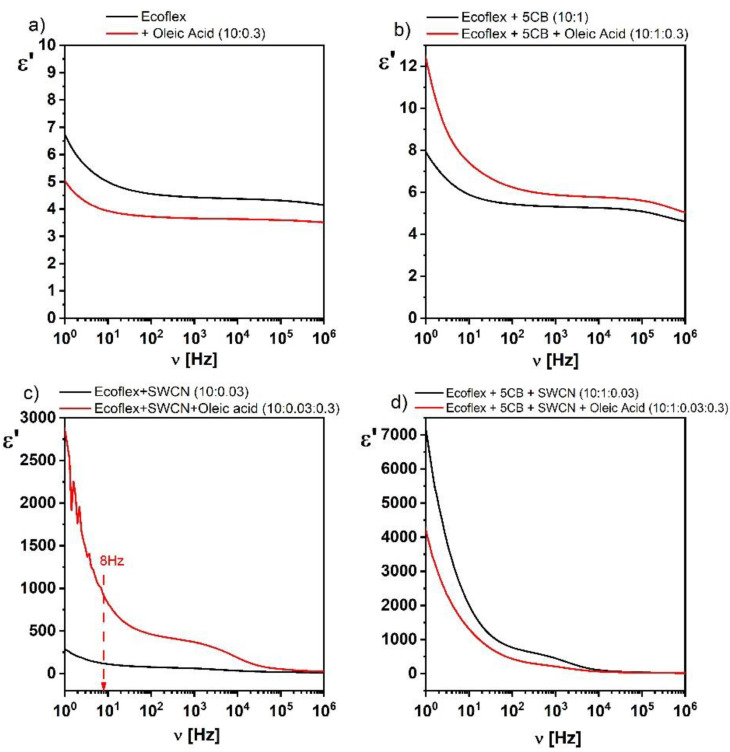
Influence of the oleic acid on the dielectric response of the pure Ecoflex^®^ (**a**), Ecoflex^®^:5CB (**b**), Ecoflex^®^:SWCN (**c**), and Ecoflex^®^:5CB:SWCN (**d**) layers.

Usually, oleic acid is used to better disperse the SWCN during sonication when preparing the hybrid layer [35,36,37]. Therefore, in this work, we decided to study the influence of oleic acid on the dielectric response of the created hybrid layers. However, we created hybrid layers only with the ratio 10:0.3 for Ecoflex:oleic acid. The effect of the presence of oleic acid on the dielectric dispersion is presented in Figure 3. As can be seen, the addition of oleic acid decreased the dielectric response of pure Ecoflex^®^ and ternary hybrid Ecoflex^®^:5CB:SWCN layers (Figure 3a,d), in contrast to the binary hybrid layers Ecoflex^®^:5CB and Ecoflex^®^:SWCN, where an increase was observed (Figure 3b,c). The influence of the oleic acid admixture was not high in the case of pure Ecoflex^®^ and Ecoflex^®^:5CB layers, in contrast to hybrid layers with the SWCN admixture (Ecoflex^®^:SWCN and Ecoflex^®^:5CB:SWCN), where this influence was significant. As shown by Wang et al. [35], this phenomenon is associated with the creation of conducting paths (percolations) in the Ecoflex^®^:SWCN layer without the admixture of oleic acid, and the contact between the SWCN is point-like. In our previous work [15], based on the analysis of the AFM images, we showed that both PCL and Ecoflex^®^ polymers willingly covered the nanotubes, making it difficult to form a conductive network. In turn, in the Ecoflex^®^:SWCN layer with the addition of oleic acid, the conductive nanotubes were insulated from each other and the matrix by an extra layer of oleic acid. As explained in [35], a chemical bond is created between the functionalized nanotubes and long chains of oleic acid, which form a monomolecular layer on the surface of the nanotubes, but in our case, there was no special procedure to guarantee the functionalization of SWCN. The contact points of the SWCN with oleic acid are equivalent to the formation of nano-capacitors, which increases the value of the dielectric constant.

Moreover, the better dispersed nanotubes with the addition of oleic acid, the greater the number of capacitors and therefore the greater the dielectric constant. As shown in Figure 3c, the value of the dielectric constant for the frequency of 100 Hz was equal to 78 and 460 for the layer without and with oleic acid addition, respectively. Thus, we observed a 6-fold increase in its value after adding oleic acid to the Ecoflex^®^:SWCN layer.

**Figure 4:**
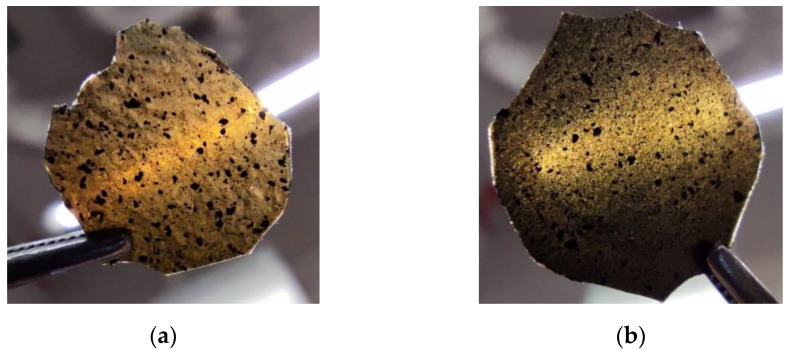
Created hybrid layers of Ecoflex^®^:SWCN (10:0.03) without (**a**) and with (**b**) the addition of oleic acid.

**Figure 5:**
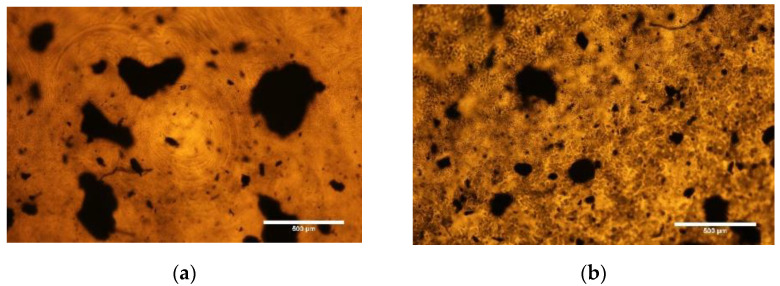
Optical textures of Ecoflex^®^:SWCN (10:0.03) hybrid layer without (**a**) and with (**b**) the addition of oleic acid.

We also found that there were some differences in the appearance of the Ecoflex^®^:SWCN hybrid layer created with and without the addition of oleic acid. As can be seen in Figure 4 and Figure 5, the number of SWCN clusters (dark spots) was much smaller, but they were bigger in the sample without oleic acid compared to the layer with the oleic acid addition. This means that the SWCN in the Ecoflex^®^:SWCN hybrid layer with oleic acid are better dispersed and can form a potentially denser conductive network, which is visible as dielectric response instability at a low frequency range (Figure 3c). The AFM topography image analysis of the Ecoflex^®^:SWCN layers with and without the addition of oleic acid (Figure S2, Table S1 in ESI) showed that the RMS was smaller for the oleic acid-doped layers, but the AFM images were different, possibly suggesting that the layers differed in composition.

In summary, the admixture of oleic acid improved the dispersion of nanotubes, however, it did not significantly change the dielectric properties of the ternary hybrid material (Ecoflex^®^:5CB:SWCN) and also did not increase the electric conductivity of the final material. Considering the different influences of oleic acid depending on the type of created layers (with 5CB or SWCN), we decided not to use oleic acid in the creation of all layers to investigate the effect of both the 5CB and SWCN dopants on the electrical properties of the hybrid layers.

## 3. Results and Discussion

**Figure 6:**
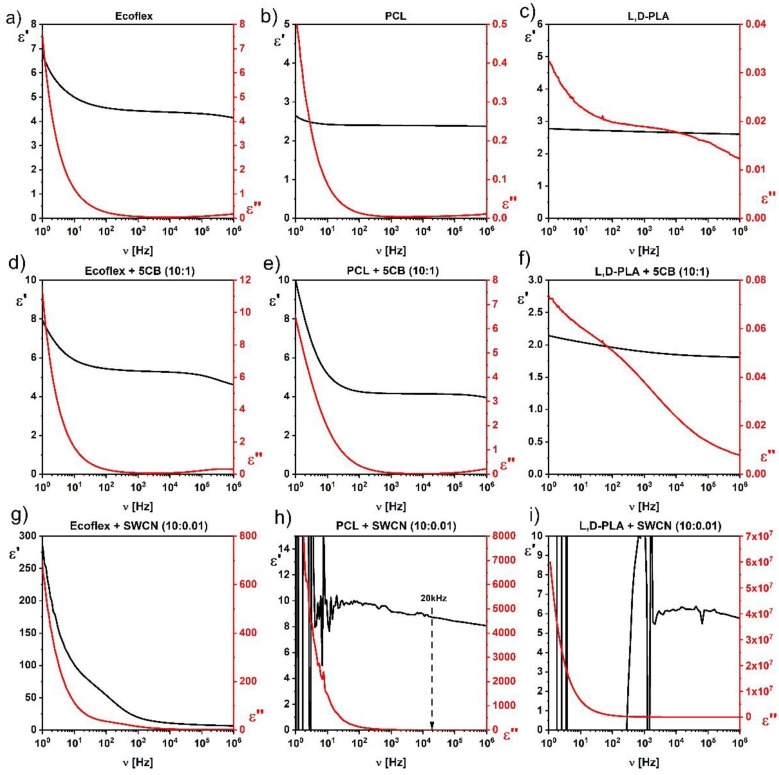
Real and imaginary part of dielectric permittivity for pure Ecoflex^®^ (**a**), PCL (**b**), L,D-PLA (**c**), with additives 5CB (**d**–**f**) and SWCN (**g**–**i**).

The influence of the liquid crystalline 5CB and conductive SWCN admixtures on the dielectric properties of the hybrid layers based on biodegradable polymers Ecoflex^®^ and PCL was studied. As seen in Figure 6, the 5CB admixture caused a slight increase in the value of the dielectric dispersion. Figure 3c shows the dielectric permittivity of the Ecoflex^®^:SWCN hybrid layer, which was about 320 at 1 Hz. This allowed us to conclude that all significant changes in the dielectric response after the addition of SWCN were due to the interaction of the nanotubes and the matrix (compare graphs in Figure 3). In turn, the dielectric permittivity of Ecoflex^®^:5CB:SWCN was about 7000 at 1 Hz (Figure 3d) and was almost 22 times higher than that without the 5CB admixture. This shows a great impact of the 5CB admixture on the dielectric properties of the created hybrid layers.

As shown in Figure 6, the addition of 5CB in a weight ratio of 10:1 increased the dielectric permittivity of the hybrid layer based on both biodegradable polymers studied (Figure 6d,e). On the other hand, the dielectric permittivity decreased for the L,D-PLA:5CB (10:1) hybrid layer in relation to pure polymer (Figure 6f), but as we know from our previous studies [13], above a certain concentration of 5CB, it increased like that observed here for the Ecoflex^®^ and PCL biodegradable polymers.

In turn, the study of the influence of SWCN on the dielectric properties of PCL and Ecoflex^®^ polymers showed that even a small amount of the SWCN admixture to the PCL matrix, making the hybrid layer conductive at low frequencies (particularly for DC) while it remained dielectric at a higher frequency region, above 20 kHz (see Figure 6h). On the other hand, the SWCN admixture increased the dielectric permittivity without any instability visible in the studied frequency range for the Ecoflex^®^ polymer matrix (Figure 6g). As we have reported in our previous papers [13,14], the addition of SWCN to the L,D-PLA polymer matrix caused the layer to become conductive, which was visible as the instability of the dielectric permittivity in the full frequency range (Figure 6i). In. summary, the SWCN admixture (in the weight ratio 10:0.01) influences the dielectric properties of PCL and L,D-PLA polymers as they become conductive (in the whole or part of the studied frequency range), while it does not change the properties of the Ecoflex^®^ layer.

**Figure 7:**
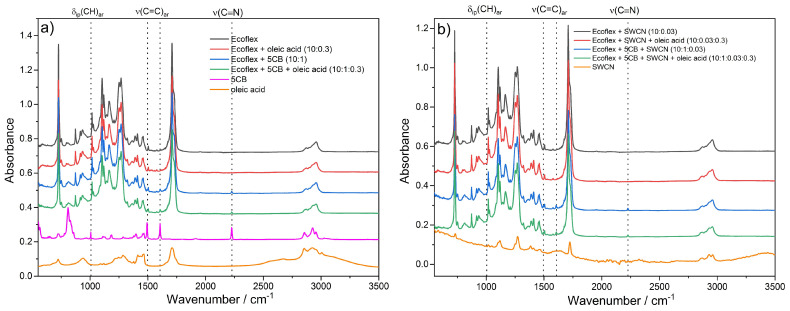
Comparison of Fourier transform infrared (FTIR) spectra of the created layers based on Ecoflex^®^ without (**a**) and with SWCN admixture (**b**).

In order to investigate the influence of SWCN, 5CB, and oleic acid on the molecular structure and chemical bonding of the studied materials, ATR FTIR spectroscopy was applied at room temperature. First of all, the spectroscopic results confirmed the proper chemical composition and no impurities in all components used in this study such as Ecoflex^®^, PCL, L,D-PLA, and 5CB. Comparing the spectra of the investigated one-, two- or three-component systems with Ecoflex^®^ before and after the addition of oleic acid no changes in positions, intensities, or widths of all bands as well as no additional new bands present in the spectra could be observed (see Figure 7). The spectra of all systems without and with oleic acid looked basically the same. This means that using this amount of oleic acid (weight ratio of Ecoflex^®^ to oleic acid = 10:0.3) did not cause any modification in the chemical bonding and molecular structure of the investigated materials. However, it is possible that hydrogen bonds can be observed between the oxygen atom of ester groups of Ecoflex^®^ and the OH group in oleic acid, as is schematically presented in the Supplement in Figure S3.

In turn, the addition of 5CB to Ecoflex^®^ and Ecoflex^®^:SWCN can be clearly seen in the spectra (Figure 7). Namely, new bands appeared at 2226, 1605, 1494, and 1006 cm^−1^. The first is connected to the ν(C≡N) stretching vibration of the nitrile group attached to the aromatic ring. The bands at 1605 and 1494 cm^−1^ corresponded to ν(C=C)_ar_ vibrations and the band at 1006 cm^−1^ to δ_ip_(CH)_ar_. Thus, these are vibrations within the biphenyl ring of 5CB, which were slightly shifted compared to the analogical vibrations of the phenyl ring in Ecoflex^®^. Surely, not all bands of 5CB are visible in the spectra of the investigated mixtures as they are overlapped by the bands connected to the main component—Ecoflex^®^. Importantly, the bands connected to 5CB, which were visible in the spectra of hybrid materials, were at exactly the same wavenumbers as in the spectrum of pure 5CB. This means that the addition of 5CB does not change the chemical bonding in the hybrid materials. In turn, the spectra of pure Ecoflex^®^ and its hybrid materials with and without SWCN were indistinguishable; the addition of SWCN was not visible and had no impact on the chemical structure of these materials.

**Figure 8:**
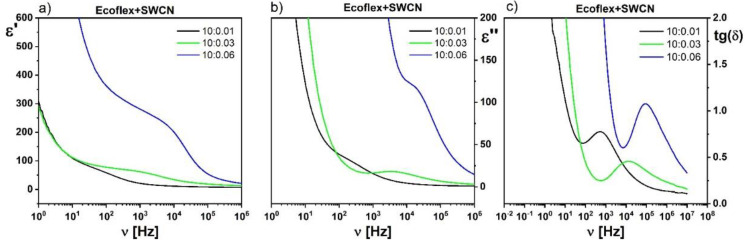
Dispersion (**a**) and absorption (**b**) of the dielectric permittivity and tangent delta (**c**) of the Ecoflex^®^:SWCN hybrid layer with different concentration of SWCN.

We also decided to study if the properties of the hybrid layer Ecoflex^®^:SWCN could be modified by changing the amount of SWCN admixture in the hybrid matrix. As above-mentioned (section: Preparation of hybrid layers), the highest concentration of SWCN in the Ecoflex^®^ matrix was a 0.06 weight ratio. Figure 8 presents the dielectric permittivity of the Ecoflex^®^:SWCN hybrid layer with different concentrations of SWCN. As can be seen, the dielectric constant increased with an increase in the content of SWCN (in the measured frequency range), but the conductive behavior (instability of the permittivity) was not visible, even for the greatest concentration at the 10:0.06 weight ratio. The Ecoflex^®^:SWCN hybrid layer remained a dielectric material. This suggests that the SWCN do not touch each other and no conducting path is formed in the polymer due to the interaction of the SWCN with the Ecoflex^®^ polymer that tightly covers them. This is in good agreement with the results presented in our recent paper [15].

**Figure 9:**
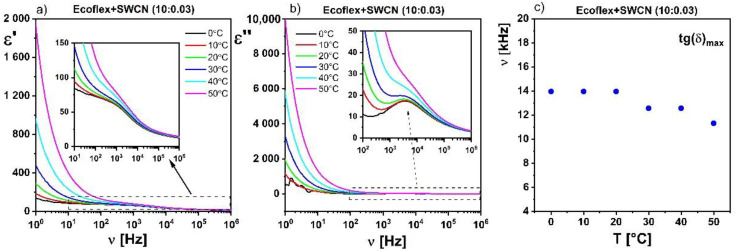
Temperature behavior of the relaxation process observed in Ecoflex^®^:SWCN (10:0.03) hybrid layer: ε’ (**a**), dielectric absorption (**b**), temperature dependence of relaxation frequency tg(δ)max (**c**).

**Figure 10:**
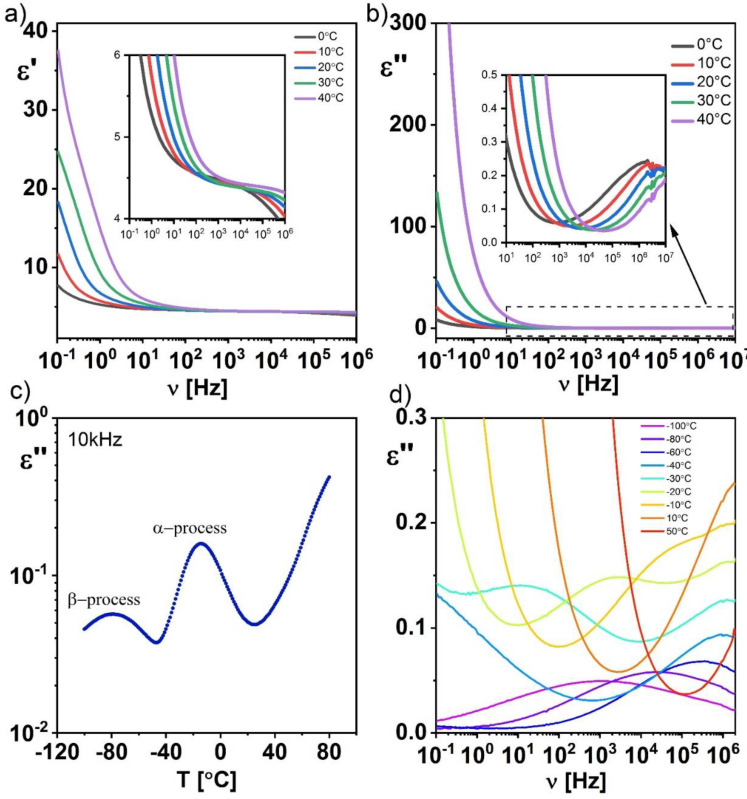
Temperature evolution of the dielectric dispersion (**a**) and absorption (**b**) as well as the dielectric absorption at 10 kHz (**c**) and for chosen temperatures (**d**) for the pure Ecoflex^®^ layer.

Moreover, it was found that after the addition of SWCN to the Ecoflex^®^ matrix, a relaxation process appeared (Figure 8), which was not visible for the pure Ecoflex^®^ layer. Its relaxation frequency as well as dielectric absorption increased with increasing SWCN admixture in the Ecoflex^®^ polymer matrix. A similar process was observed for MWCN with PANI hybrid systems in a polystyrene matrix and was identified as conduction relaxation [38]. Temperature dependence of the dielectric response of the Ecoflex^®^:SWCN hybrid layer was also studied. As an example, the temperature behavior of this process is presented for a concentration of 10:0.03 in Figure 9. As can be seen, this process, visible at 5 kHz, is temperature independent (Figure 9c). The same behavior was observed for the other concentrations. It should be stressed here that this process did not exist for the pure Ecoflex^®^ matrix (see Figure 10), only for the hybrid layers with the SWCN admixture. Moreover, it was not visible for the other two polymers studied (PCL and L,D-PLA) because even a small admixture of SWCN made the hybrid layers conductive in those cases.

For the pure Ecoflex^®^ polymer layer, two commonly known [39] relaxation processes were registered in the studied frequency and temperature range (Figure 10): the β-process at low temperatures and the α-process at temperatures above −40 °C. Both processes were observed simultaneously at −30 °C and merged at higher temperature (Figure 10d). At room temperature, which is important from an application point of view, only the α-process is visible. This process occurs around the glass transition temperature (Tg = −33 °C for Ecoflex^®^ as confirmed by the dielectric spectra, see Figure 10d) and is one of the determinants of the α-process [40]. The relaxation frequency of both these processes (α and β) increased with increasing temperature in contrast to the temperature-independent conduction relaxation registered in the Ecoflex^®^:SWCN hybrid layer (Figure 10c).

Based on the permittivity measurements, the estimated refractive index *n*’ was calculated [41] for all created layers according to Equation (1) and the results are presented in Table 1.

(1) $$n^{\prime }=\frac{1}{\sqrt{2}}\sqrt{\sqrt{\varepsilon^{\prime 2}+\varepsilon^{\prime \prime 2}}+\varepsilon^{\prime }}$$

As can be seen, the estimated refractive index changes depending on the dopant. The addition of 5CB to the PCL and Ecoflex^®^ layers caused an increase in the estimated refractive index, while for the L,D-PLA layer, it caused its reduction. On the other hand, the addition of SWCN always caused an increase in the refractive index, which increased with an increasing concentration of SWCN, as shown for the Ecoflex^®^ layer. Furthermore, the admixture of oleic acid to the Ecoflex^®^ and Ecoflex^®^:5CB:SWCN layers reduced the estimated refractive index, and its increase in the Ecoflex^®^:SWCN and Ecoflex^®^:5CB layers.

**Figure 11:**
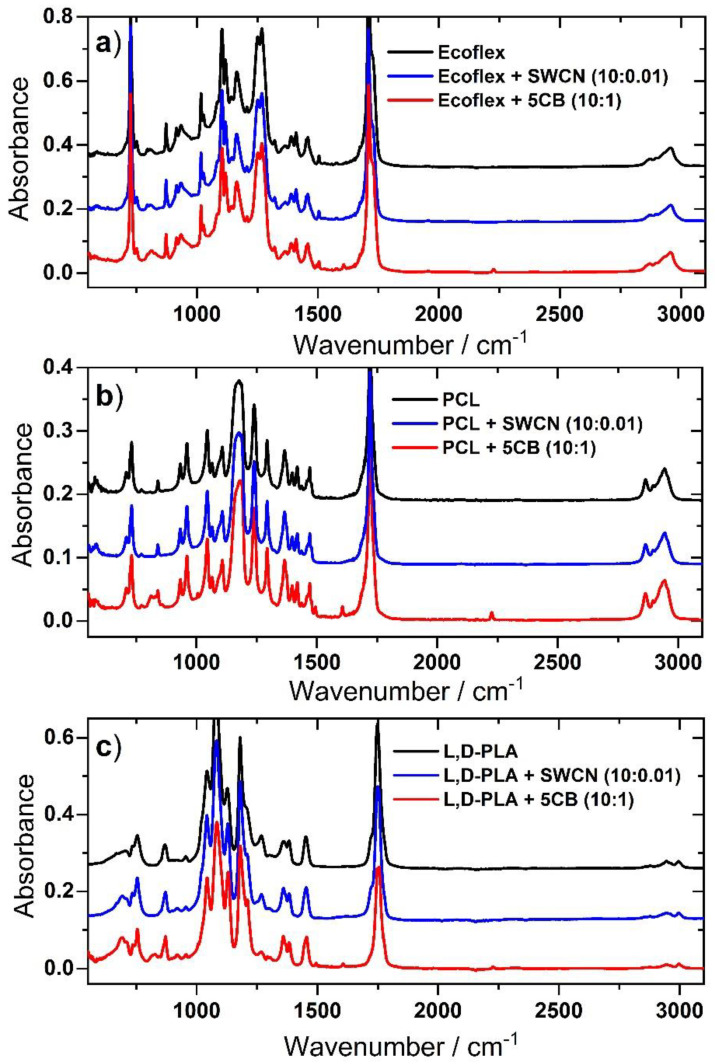
Comparison of FTIR spectra of Ecoflex^®^ (**a**), PCL (**b**), and L,D-PLA (**c**) layers without and with the addition of SWCN or 5CB.

The IR spectra of the pure polymer matrixes: Ecoflex^®^, PCL, and L,D-PLA and with the addition of SWCN or 5CB in the weight ratio of 10:0.01 and 10:1, respectively, are presented in Figure 11. In all three cases, the spectra of pure polymer and with the addition of SWCN look basically the same. Thus, this amount of carbon nanotubes does not cause molecular interactions with the polymer matrix. It should be noted, however, that the increase in the amount of SWCN may affect the results of spectroscopic studies, but this was not the topic of our work. Moreover, as we have shown in our previous work [13], as the concentration of SWCN in the polymer layer increased, the transparency of the layer decreased and such a hybrid layer was not suitable for use as a transparent electrode in flexible devices.

In turn, the addition of 5CB was clearly visible in all spectra of the three polymers in the form of new bands at about 2226, 1605, 1494, and 1006 cm^−1^. In the case of PCL:5CB, additional new bands also became apparent at 829 and 856 cm^−1^. These bands were connected to out of plane bending vibrations of the biphenyl ring in 5CB. In the case of Ecoflex^®^:5CB and PCL:5CB, the admixture was present in the spectra, however, it did not cause any change in the appearance of the bands connected to the two polymers used.

The situation was different for the L,D-PLA:5CB system. In addition to new bands associated strictly to 5CB, some changes in the spectral regions, where the bands connected to L,D-PLA appeared, were evident. The band at 1748 cm^−1^ connected to the ν(C=O) stretching vibration of the carbonyl group shifted by 3 cm^−1^ toward larger wavenumbers and split into at least three components with a maxima at about 1748, 1750, and 1755 cm^−1^. At the same time, the shoulder at 1722 cm^−1^, visible in pure L,D-PLA, did not appear in the hybrid material. An increase in intensity and shift toward larger wavenumbers could be noticed for the bands at 1127 and 1081 cm^−1^, respectively, both connected to the ν(C-O) vibrations. Furthermore, the bands at 1382 and 1360 cm^−1^ associated with δ(CH_3_) vibrations split into two components, whereas both bands at 869 and 754 cm^−1^ associated with ν(C–C) vibrations became much narrower. Additionally, in the spectrum of L,D-PLA:5CB, two new bands could be seen at ~920 and 690 cm^−1^. Importantly, the band at 2226 cm^−1^, connected to ν(CN) vibration of 5CB, was split in the spectrum of the hybrid material; an additional shoulder was observed at 2232 cm^−1^. It is clear that all molecular groups of L,D-PLA are influenced by the addition of the 5CB additive and that bonding interactions most likely occur between the carbonyl groups of L,D-PLA and nitrile groups of 5CB.

## 4. Conclusions

The dielectric properties of hybrid binary and ternary composites based on biodegradable polymers (Ecoflex^®^ and PLA) and SWCN and/or liquid crystalline compound 5CB were studied. The results were compared with our previous results obtained for the L,D-PLA polymer [13,15]. It was found that while the admixture of oleic acid improved the dispersion of nanotubes, no influence on the molecular structure of hybrid materials based on Ecoflex^®^ polymer matrix was observed in the IR spectra as well as on the dielectric response. Each of the pure polymers studied is a quite a good candidate for use in electronic components where a biodegradable dielectric material is needed. We show in detail the dielectric properties of the created hybrid materials, and the following conclusions can be formulated:

1. As is known, the materials useful in electronics require small dielectric losses. Ecoflex^®^ exhibited significant dielectric losses, especially at low frequencies, in contrast to the L,D-PLA and PCL polymers. However, due to other advantages (i.e., very good adhesion to nanotubes), Ecoflex^®^ cannot be discredited and can find applications where this behavior is desired.
2. The cut-off frequency below which the hybrid material becomes conductive was determined by the dielectric spectroscopy method and in the case of the PCL:SWCN (10:0.01) composite, it was set at 20 kHz. In turn, the L,D-PLA:SWCN (10:0.01) hybrid material was much more conductive while the composites based on the Ecoflex^®^ polymer became stable in the whole measuring frequency range, but with very high dielectric response.
3. At room temperature range, important from an application point of view, one relaxation process was registered for the pure Ecoflex^®^ polymer layer, namely, the α-process. The second process, the β-process, was found at low temperatures. The relaxation frequency of both processes increases with increasing temperature.
4. After the addition of the SWCN to the Ecoflex^®^ polymer matrix, it remained a dielectric and the temperature independent conduction relaxation process was visible in the dielectric spectra of the created binary layers, while the binary hybrid layers PCL:SWCN and L,D-PLA:SWCN (in a weight ratio of 10:0.01) became conductive.
5. From the point of view of infrared absorption spectroscopy, no chemical interactions between the investigated polymers and additives (SWCN, 5CB) were observed except for the L,D-PLA:5CB hybrid layer. In this system, the 5CB additive moderates the L,D-PLA matrix.

In summary, among these three biodegradable polymers (Ecoflex^®^, PCL, and L,D-PLA) studied for electronic applications, hybrid materials based on the polymer L,D-PLA still seem to be the best choice as they exhibited the highest possible electrical conductivity and the lowest possible energy losses.

## Figures and Tables

**Table 1:** Estimated refractive index *n*′ at 1 MHz.

Created Layer	$\varepsilon^{\prime }$	$\varepsilon^{\prime \prime }$	$n^{\prime }$
PCL	2.37	0.01	1.54
PCL + SWCN (10:0.01)	8.06	0.30	2.84
PCL + 5CB (10:1)	3.94	0.22	1.98
L,D-PLA	2.60	0.01	1.61
L,D-PLA + SWCN (10:0.01)	5.72	46.64	5.13
L,D-PLA + 5CB (10:1)	1.81	0.01	1.34
Ecoflex^®^	4.14	0.17	2.04
Ecoflex^®^ + SWCN (10:0.01)	6.47	0.89	2.55
Ecoflex^®^ + SWCN (10:0.02)	8.22	1.76	2.88
Ecoflex^®^ + SWCN (10:0.03)	13.22	3.09	3.66
Ecoflex^®^ + SWCN (10:0.06)	21.33	14.56	4.86
Ecoflex^®^ + 5CB (10:1)	4.61	0.31	2.15
Ecoflex^®^ + 5CB +SWCN (10:1:0.03)	19.51	9.41	4.54
Ecoflex^®^ + Oleic Acid	3.51	0.11	1.87
Ecoflex^®^ + 5CB + Oleic Acid (10:1:0.3)	5.04	0.42	2.25
Ecoflex^®^ + SWCN + Oleic Acid (10:0.03:0.3)	24.51	13.73	5.13
Ecoflex^®^ + 5CB +SWCN + Oleic Acid (10:1:0.03:0.3)	15.63	5.97	4.02

## Data Availability

The data presented in this study are available in Supplementary Materials and on request from the corresponding author.

## Supplementary Materials

The following are available online at https://www.mdpi.com/article/10.3390/ma14071719/s1, Figure S1: Photos of created hybrid layers: Ecoflex^®^:SWCN (10:0.02) (a), Ecoflex^®^:SWCN (10:0.03) (b), Ecoflex^®^:SWCN (10:0.06) (c), Ecoflex^®^:5CB:SWCN (10:1:0.03) (d), Ecoflex^®^:Oleic Acid (10:0.3) (e), Ecoflex^®^:5CB:Oleic Acid (10:1:0.3) (f), Ecoflex^®^:SWCN:Oleic Acid (10:0.03:0.3) (g), Ecoflex^®^:5CB:SWCN:Oleic Acid (10:1:0.03:0.3) (h), Figure S2: AFM images for Ecoflex^®^+SWCN (10:0.03) (a,c,e) and Ecoflex^®^+SWCN+Oleic Acid (10:0.03:0.3) (b,d,f) layers at different scales, Figure S3: Scheme of possible hydrogen bonds interactions between Ecoflex^®^ and oleic acid. In our study, however, the amount of oleic acid (ca. 3% mass) in the investigated systems did not cause such interactions – the chemical structure of Ecoflex^®^ was not affected, Table S1: Statistics parameter of AFM images presented in Figure S2.
