# Supplementary material for: Comparison of the Dielectric Properties of Ecoflex® with L,D-Poly(Lactic Acid) or Polycaprolactone in the Presence of SWCN or 5CB
Source: Materials (Basel). 2021 Mar 31;14(7):1719. doi: 10.3390/ma14071719 (PMC8037482; doi:10.3390/ma14071719)
Supplement: Supplementary file 1 [file materials-14-01719-s001.pdf]

materials

MDPI

Article

Comparison of the Dielectric Properties of Ecoflex® with L,D-Poly(Lactic Acid) or Polycaprolactone in the Presence of SWCN or 5CB

Patryk Fryń [1], Sebastian Lalik [1], Natalia Górska [2], Agnieszka Iwan [3,*] and Monika Marzec [1,*]

1 Institute of Physics, Jagiellonian University, S. Lojasiewicza 11, 30-348 Krakow, Poland; patryk.fryn@doctoral.uj.edu.pl (P.F.); sebastian.lalik@doctoral.uj.edu.pl (S.L.)
2 Faculty of Chemistry, Jagiellonian University, Gronostajowa 2, 30-387 Krakow, Poland; gorska@chemia.uj.edu.pl
3 Faculty of Security and Safety Research, General Tadeusz Kosciuszko Military University of Land Forces, Czajkowskiego 109 Str., 51-147 Wroclaw, Poland
* Correspondence: agnieszka.iwan@awl.edu.pl (A.I.); monika.marzec@uj.edu.pl (M.M.)

Citation: Fryń, P.; Lalik, S.; Górska, N.; Iwan, A.; Marzec, M. Comparison of the Dielectric Properties of Ecoflex® with L,D-Poly(Lactic Acid) or Polycaprolactone in the Presence of SWCN or 5CB. *Materials* **2021**, *14*, 1719. https://doi.org/10.3390/ma14071719

Academic Editor: Christoforos Krontiras

Received: 5 March 2021
Accepted: 29 March 2021
Published: 31 March 2021

Publisher's Note: MDPI stays neutral with regard to jurisdictional claims in published maps and institutional affiliations.

Copyright: © 2021 by the authors. Licensee MDPI, Basel, Switzerland. This article is an open access article distributed under the terms and conditions of the Creative Commons Attribution (CC BY) license (http://creativecommons.org/licenses/by/4.0/).

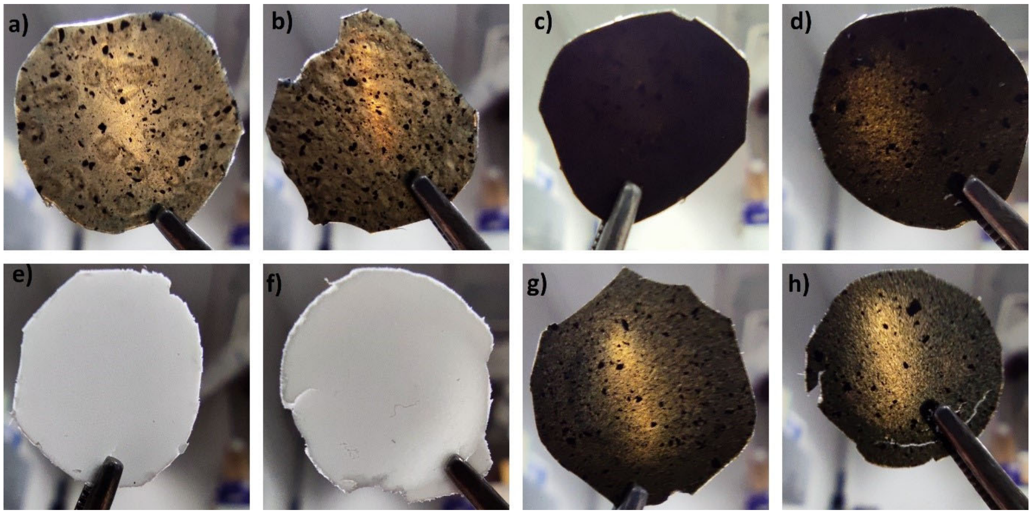

Figure S1. Photos of created hybrid layers: Ecoflex®:SWCN (10:0.02) (**a**), Ecoflex®:SWCN (10:0.03) (**b**), Ecoflex®:SWCN (10:0.06) (**c**), Ecoflex®:5CB:SWCN (10:1:0.03) (**d**), Ecoflex®:Oleic Acid (10:0.3) (**e**), Ecoflex®:5CB:Oleic Acid (10:1:0.3) (**f**), Ecoflex®:SWCN:Oleic Acid (10:0.03:0.3) (**g**), Ecoflex®:5CB:SWCN:Oleic Acid (10:1:0.03:0.3) (**h**).

AFM images were done at room temperature by using the Agilent 5500 microscope working in non-contact mode. Parameters such as setpoint, proportional gain, integral gain and speed were adjusted to each measurement to obtain the best possible images. Topography images were collected at several randomly chosen areas for each sample and for different magnification to make sure that presented results are reliable. Image processing was done using the Gwyddion programme.

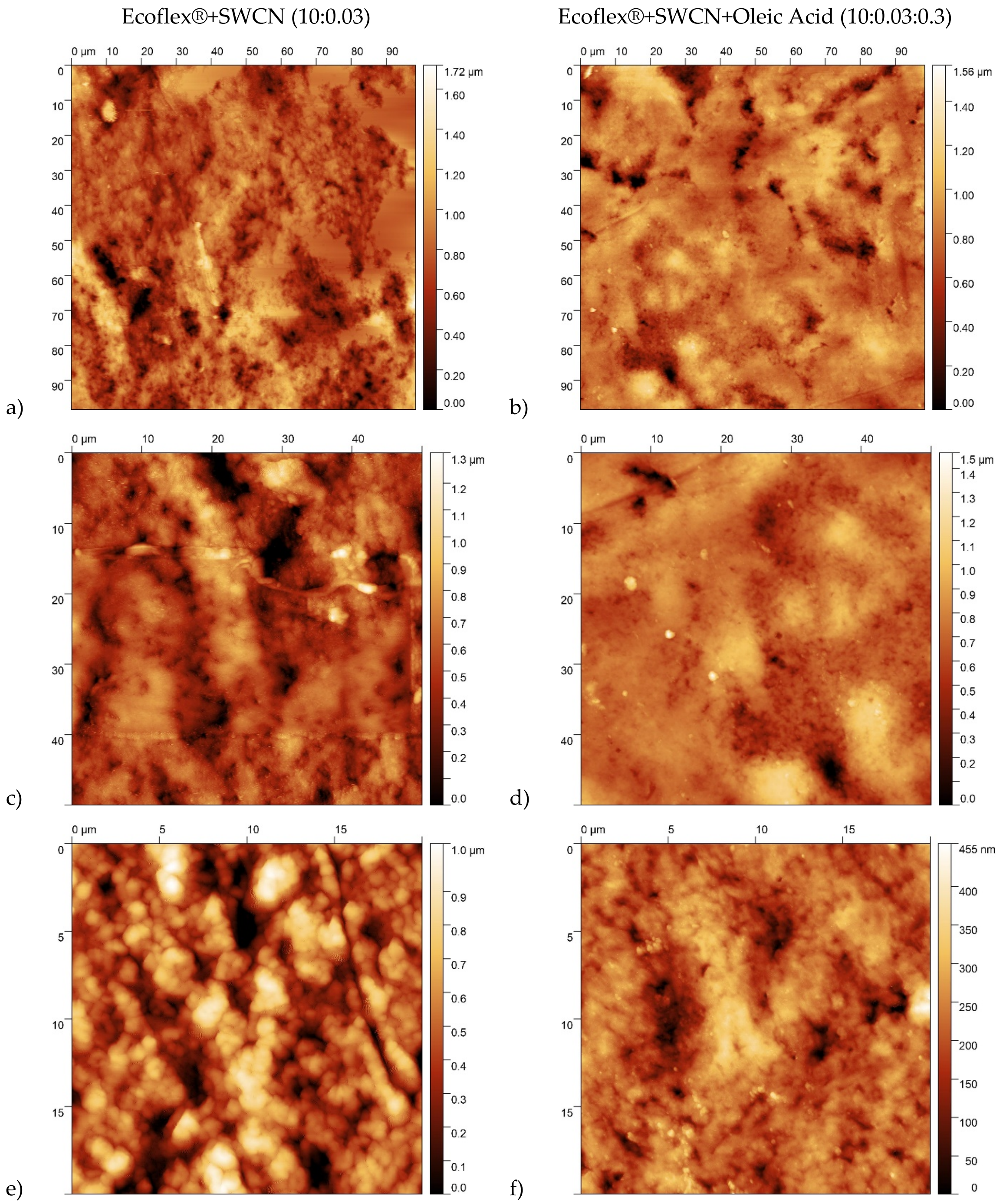

Figure S2. AFM images for Ecoflex®+SWCN (10:0.03) (**a**,**c**,**e**) and Ecoflex®+SWCN+Oleic Acid (10:0.03:0.3) (**b**,**d**,**f**) layers at different scales.

Table 1. Statistics parameter of AFM images presented in Figure S2.

Created Layer	Ecoflex®+SWCN (10:0.03)			Ecoflex®+SWCN+Oleic Acid (10:0.03:0.3)		
Image size [µm]	100 × 100	50 × 50	20 × 20	100 × 100	50 × 50	20 × 20
Average value [nm]	738.07	503.44	460.19	771.84	709.96	210.92
RMS roughness (Sq) [nm]	209.76	175.89	191.41	170.28	143.42	54.97
Mean roughness (Sa) [nm]	162.65	136.48	151.78	125.20	105.99	42.24
Skew (Ssk)	-0.20	-0.16	0.05	-0.94	-0.36	-0.35
Excess kurtosis	0.56	0.59	-0.13	2.21	2.22	0.83
Minimum [µm]	0.00	0.00	0.00	0.00	0.00	0.00
Maximum [µm]	1.72	1.31	1.04	1.56	1.46	0.46
Median [µm]	0.75	0.51	0.46	0.79	0.71	0.21
Maximum peak height (Sp) [µm]	0.98	0.81	0.58	0.79	0.75	0.24
Maximum pit depth (Sv) [µm]	0.74	0.50	0.46	0.77	0.71	0.21
Maximum height (Sz) [µm]	1.72	1.31	1.04	1.56	1.46	0.46

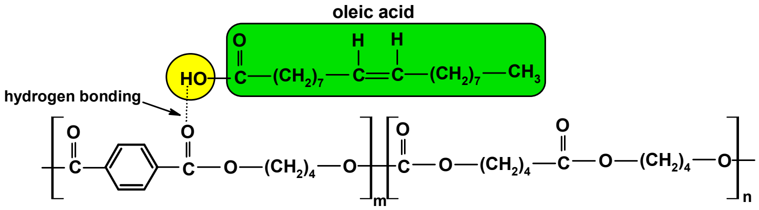

Figure S3. Scheme of possible hydrogen bonds interactions between Ecoflex® and oleic acid. In our study, however, the amount of oleic acid (ca. 3% mass) in the investigated systems did not cause such interactions – the chemical structure of Ecoflex® was not affected.
